# Secreted protein acidic and rich in cysteine (SPARC) is associated with nasopharyngeal carcinoma metastasis and poor prognosis

**DOI:** 10.1186/1479-5876-10-27

**Published:** 2012-02-09

**Authors:** Hai-Yun Wang, Yang-Yang Li, Qiong Shao, Jing-Hui Hou, Fang Wang, Man-Bo Cai, Yi-Xin Zeng, Jian-Yong Shao

**Affiliations:** 1 State Key Laboratory of Oncology in Southern China, Sun Yat-Sen University Cancer Center, 651 Dong Feng Road East, Guangzhou 510060, People's Republic of China; 2 Department of Molecular Diagnostics, Sun Yat-Sen University Cancer Center, 651 Dong Feng Road East, Guangzhou 510060, People's Republic of China; 3 Department of Pathology, Sun Yat-Sen University Cancer Center, 651 Dong Feng Road East, Guangzhou 510060, People's Republic of China; 4 Department of Experiment Research, Sun Yat-Sen University Cancer Center, 651 Dong Feng Road East, Guangzhou 510060, People's Republic of China

**Keywords:** SPARC, Nasopharyngeal carcinoma, Metastasis

## Abstract

**Background:**

The aim of the present study was to analyse the expression of Secreted protein acidic and rich in cysteine (SPARC) in nasopharyngeal carcinoma (NPC) specimens, and to evaluate its correlation with clinicopathologic features, including survival of patients with NPC

**Methods:**

NPC tissue microarrays (TMAs) were constructed from Sun Yat-sen University Cancer Center (SYSUCC), another three centers on mainland China, Singapore and Hong Kong. Using quantitative RT-PCR and Western-blotting techniques, we detected mRNA and protein expression of SPARC in NPC cell lines and immortalized nasopharyngeal epithelial cells (NPECs) induced by Bmi-1 (NPEC2 Bmi-1). The difference of SPARC expression in the cell lines was tested using a *t*-test method. The relationship between the SPARC expression and clinicopathological data was assessed by chi-square. Survival analysis was estimated using the Kaplan-Meier approach with log-rank test. Univariate and multivariate analyses of clinical variables were performed using Cox proportional hazards regression models.

**Results:**

The expression levels of SPARC mRNA and protein were markedly higher in NPC cell lines than in NPEC2 Bmi-1. Especially, the expression levels of SPARC mRNA and protein were much lower in the 6-10B than in the 5-8 F (*P *= 0.002, *P *= 0.001). SPARC immunostaining revealed cytoplasmic localization in NPC cells and no staining in the stroma and epithelium.

In addition, high level of SPARC positively correlated with the status of distant metastasis (*P *= 0.001) and WHO histological classification (*P *= 0.023). NPC patients with high SPARC expression also had a significantly poorer prognosis than patients with low SPARC expression (log-rank test, *P *< 0.001), especially patients with advanced stage disease (log-rank, *P *< 0.001). Multivariate analysis suggested that the level of SPARC expression was an independent prognostic indicator for the overall survival of patients with NPC (*P *< 0.001).

**Conclusions:**

SPARC expression is common in NPC patients. Our data shows that elevated SPARC expression is a potential unfavorable prognostic factor for patients with NPC.

## Background

Nasopharyngeal carcinoma (NPC) is unique amongst head and neck cancers because of its peculiar epidemiological and biological characteristics. NPC is a rare tumor in most parts of the world, but it occurs at a high rate in Southeast Asia. Unlike other head and neck malignancies, NPC is notorious for its highly metastatic nature [1]. Metastasis to regional lymph nodes or distant organs, and local recurrence, are two major causes for treatment failure of this cancer. Although NPC is classified as a subtype of head and neck squamous cell carcinoma, its unique epidemiology, clinical characteristics, etiology, and histopathology warrant separate efforts for the study of its underlying molecular mechanisms of carcinogenesis [2]. For example, NPC patients tend to present at a more advanced stage of disease because the primary anatomical site of tumor growth is located in a silent area, and they exhibit higher metastatic potential when compared to other head and neck squamous cell carcinoma [3-5].

Currently, the prediction of NPC prognosis is mainly based on clinical (Tumor, Node, Metastasis) TNM staging. However, NPC patients with the same clinical stage often present different clinical outcomes, suggesting that TNM staging is insufficient for precisely predicting the prognosis of this disease [6-9]. The specific genetic changes underlying the development and progression of this neoplasm are not completely understood. Therefore, the identification of useful biomarkers associated with NPC holds the promise of improved clinical management.

Secreted protein acidic and rich in cysteine (SPARC), also known as osteonectin or BM-40, is a matricellular glycoprotein that functions primarily to promote extracellular matrix deposition [10]. It is expressed at high levels in bone tissues and is widely distributed in many other tissues and cell types [11]. Originally detected as a component of bone, it is now known to be expressed at high levels in tissues undergoing mineralization, proliferation, and re-modeling, as well as in a wide range of malignancies [12].

High SPARC expression in primary tumors, including gastric cancer, correlates with metastasis and poor prognosis [13,14]. Elevated mRNA level in tumor tissue is associated with a poorer survival in breast cancer [15-17], osteosarcoma [18], glioblastoma [19], oesophageal carcinoma [20], and bladder cancer [21]. Immunohistochemical detection of SPARC protein in tumor cells is associated with survival in meningiomas [22], tongue carcinoma [23], head and neck cancer [24] and cutaneous malignant melanomas [25]. Interestingly, in pancreatic adenocarcinoma [26,27] and non-small cell lung cancer [28], only SPARC expression in peritumoural stroma is associated with survival. The possible clinical significance of SPARC expression has remained unclear in NPC patients.

In this study, we first investigated the clinical variables of SPARC expression in NPC patients from different institutions. Using quantitative RT-PCR and Western blot analysis, we detected mRNA and protein expression of SPARC in NPC cell lines, and immortalized nasopharyngeal epithelial cells (NPECs) induced by Bmi-1 (NPEC2 Bmi-1). Immunohistochemistry (IHC) on TMAs was used to assess SPARC expression in NPC tissue from three cities in mainland China, as well as Hong Kong and Singapore. Then, the relationship between SPARC expression and NPC patients' prognosis was investigated. Overall, our findings indicate that high SPARC expression may serve as an independent prognostic marker for predicting poor prognosis in NPC patients, especially those with advanced stage disease.

## Methods

### Samples and cases

For this retrospective study, enrolled NPC cases included a cohort of 836 patients with incident, primary, biopsy-confirmed NPC who were diagnosed between 1992 and 2002 at SYSUCC (Guangzhou, China); a cohort of 132 patients with incident, primary, biopsy-confirmed NPC who were diagnosed between 1992 and 2004 from three other cities in mainland China; and a cohort of 125 patients with biopsy-confirmed NPC who were diagnosed between 2002 and 2004 at cancer centers in Hong Kong and Singapore. The clinicopathological characteristics are summarized in Table 1. Inclusion criteria were: availability of hematoxylin and eosin (H&E) slides with invasive tumor components, treatment before the end of 2005, availability of follow-up data, no history of treated cancer, and appropriate patient informed consent. Cancer TNM stage was defined according to the 1992 China Staging system for cases from mainland China (*n *= 968), and the 1997 American Joint Committee on Cancer staging system [29,30] for cases from Hong Kong and Singapore (*n *= 125). All patients underwent standard curative radiotherapy with or without chemotherapy. Institute Research Medical Ethics Committee of SYSUCC granted approval for this study.

**Table 1:** Clinicopathological characteristics and follow-up data of 1093 NPC patients

Characteristic	Number of patients (%)
**Sex**	
Female	318 (29.1)
Male	775 (70.9)
**Age (years)**	
Median	
(range)	47 (15-90)
≤ 47	568
> 47	517
Missing	8
**Follow-up time (months)**	
Median	67 (1-120)
(range)	
**Clinical stage**	
I-II	315 (29.0)
III-IV	773 (71.0)
Missing	5
**Relapse**	
No	1001 (91.7)
Yes	91 (8.3)
Missing	1
**Metastasis**	
No	984 (90.1)
Yes	108 (9.9)
Missing	1
**Therapeutic modality**	
Radiotherapy	766 (70.6)
Chemotherapy	10 (0.9)
Radiochemotherapy	309 (28.5)
Missing	8
**WHO histological classification**	
NKUC	853 (78.3)
NKDC	206 (18.9)
KSCC	30 (2.8)
Missing	4

OS rate (%)	
5-year	69.1

Abbreviations: *NPC *nasopharyngeal carcinoma; *WHO *World Health Organization; *NKUC *non-keratinizing undifferentiated carcinoma; *NKDC *non-keratinizing differentiated carcinoma; *KSCC *keratinizing squamous cell carcinoma; *OS *overall survival

### Tissue microarrays (TMAs) construction

A fresh section stained with hematoxylin and eosin (HE) was cut from each block. Individual donor blocks were overlaid with the corresponding HE slides, and areas for TMA sampling were marked. Using instrumentation developed at the Mayo Clinic (Beech Instrument Co., USA), two cylindrical cores of 1.0 mm at their greatest dimension were removed from each donor paraffin block and transferred to pre-molded recipient paraffin blocks at defined array positions. Recipient paraffin blocks contained holes of appropriate dimension in a grid pattern of a maximum of 11 holes in width by 14 holes in length, allowing for 154 tissue cores per block. This design permitted multiple blocks with identical array patterns to be constructed simultaneously, serially sectioned at 4 μm onto "charged" glass slides, and stored at 4°C.

### Immunohistochemistry staining

Immunohistochemistry (IHC) staining was performed using TMA sections that were rehydrated through a graded alcohol series. Endogenous peroxidase activity was blocked with 3% hydrogen peroxide for 10 min at room temperature. For antigen retrieval, TMA slides were boiled in tris (hydroxymethyl) aminomethane-ethylenediaminetetraacetic acid buffer (pH 8.0) in a pressure cooker for 2 min, 30 sec. TMA slides were incubated with anti-SPARC (1:100 dilution; Abnova Laboratories, USA), in a moist chamber overnight at 4°C. The next day, slides were treated by HRP polymer conjugated anti-mouse secondary antibody (Dako, Glostrup, Denmark) for 30 min at 37°C, followed by a 3 min incubation in diaminobenzidine (DAB) solution for protein detection. The nucleus was counterstained with Meyer's hematoxylin. A negative control was obtained by replacing the primary antibody with normal murine IgG.

### Assessment of immunostaining

Immunostaining results were evaluated and scored independently by two pathologists lacking knowledge of the clinicopathological outcomes of the patients. SPARC staining results were scored as four levels according to the percentage of cytoplasmic positive tumor cells in 10 high power fields as follows [31]. (-): less than 5%; (+): 6%-25%; (++): 26-50%; (+++): more than 50%. Likewise, staining intensity was assigned a score as follows: 0 = no staining; 1 = weak staining; 2 = moderate staining; 3 = strong staining. The two individual parameters were added [32], resulting in an immunoreactivity score (IRS) ranging from 0 to 6. We defined cases with IRS > 4 as high expression, and cases with IRS ≤ 4 as low expression [32].

### Cell lines and cell cultures

Immortalized NPECs induced by Bmi-1 (NPEC2 Bmi-1) were established as described previously [33] and grown in keratinocyte/serum-free medium (Invitrogen). The human NPC cell lines CNE1, CNE2, HONE1, SUNE1, 5-8 F, and 6-10B were incubated in RPMI-1640 medium supplemented with 10% fetal bovine serum (FBS) (Gibco, USA), 100 units of penicillin/ml and 100 μg of streptomycin/ml. The human NPC cell line C666 was cultured in RPMI 1640 medium (Gibco, USA) containing 15% FBS. All cell lines were maintained in a humidified incubator at 37°C with 5% CO_2_.

### RNA extraction and quantitative RT-PCR analysis of SPARC

Total RNA from 7 NPC cell lines and NPEC2 Bmi-1 were isolated using Trizol reagent ((Life Technologies, Grand Island, NY) according to the manufacturer's instructions. RNA concentrations were determined with NanoDrop (NanoDrop Technologies, Inc.). Following the manufacturer's instructions, cDNA was prepared from 2 ug total RNA by TaKaRa reagent and amplified using SYBR Green chemistry (Invitrogen) on an ABI 7500HT instrument (ABI Inc., USA). The following primers were used: SPARC forward 5'-GTGCAGAGGAAACCGAAGAG-3';SPARC reverse 5'-TCATTGCTGCACACCTTCTC-3'; GAPDH forward 5'-CTGCACCACCAACTGCTTAG-3';GAPDH reverse 5'AGGTCCACCACTGACACGTT-3'. After 40 cycles, data reduction was performed with Sequence Detection System Software (Applied Biosystems Inc.,). For data analysis, threshold cycles (Ct) for GAPDH (reference) and SPARC (sample) were determined in triplicates (shown as arithmetical mean). The quantity of SPARC in each NPC cell line relative to the average expression in NPEC2 Bmi-1 cell line, was calculated using the equation: RQ = 2-^ΔΔ^CT [34].

### Western blotting analysis

Equal amounts of whole-cell lysates were resolved by sodium dodecyl sulfate polyacrylamide gel electrophoresis (SDS-PAGE) and transferred to a polyvinylidene difluoride (PVDF) membrane (Pall Corp., Port Washington, NY). This was followed by incubation with primary mouse monoclonal antibodies against human SPARC (1:100 dilution; Abnova), and mouse monoclonal antibodies against human GAPDH (1:4000 dilution; Santa Cruz Biotechnology, Santa Cruz, CA), respectively. Immunoreactive proteins were detected with enhanced chemiluminescencedetection reagents (Amersham Biosciences, Uppsala, Sweden) according to the manufacturer's instructions.

### Statistical analysis

Data was analyzed using SPSS software, version 16.0 (SPSS Inc., Chicago, USA). The difference of means (SPARC expression in the NPEC2 Bmi-1 and NPC cell lines) was tested using a *t*-test method. The correlation between SPARC expression and clinicopathological parameters was assessed by chi-square test. Kaplan-Meier analysis and log-rank test were used to assess survival rate, and to compare survival rate differences. Univariate and multivariate regression analysis were performed with the Cox proportional hazards regression model to analyse the factors related to prognosis. A *P*-value less than 0.05 was considered as statistically significant.

## Results

### SPARC expression in NPC cell lines and tissue

**Figure 1:**
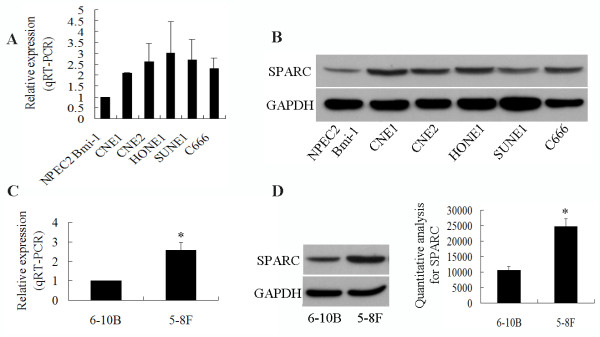
**Expression of SPARC in cell lines**. (A) Quantitative RT-PCR detection showed that NPC cell lines of CNE1, CNE2, HONE1, SUNE1 and C666 presented higher level of mRNA expression of SPARC than that in NPEC2 Bmi-1. (B) Western blot analysis showed that the expression of SPARC protein levels in NPC cell lines was higher than that in NPEC2 Bmi-1. (C) Quantitative RT-PCR detection showed that 5-8 F presented higher level of mRNA expression of SPARC than that in 6-10B (*P *= 0.002). (D) Western blot analysis showed that the expression of SPARC protein levels in 5-8 F was higher than that in 6-10B (*P *= 0.001). Results are shown as expression relative to GAPDH and are means (± SD) of 3 experiments.

**Figure 2:**
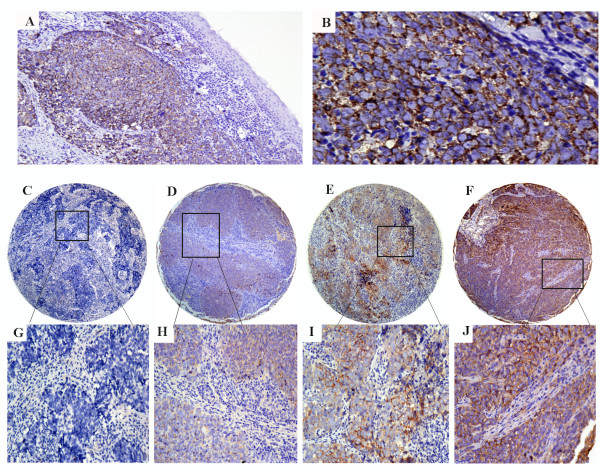
**Representative staining of SPARC in NPC tissue by immunohistochemistry**. A (100×) and B (400 ×) showing the expression of SPARC detection by IHC in NPC tumors and nasopharyngeal epithelium.; (C) no staining of SPARC in NPC tissue by immunohistochemistry; (D) weak staining in cytoplasm; (E) moderate staining in cytoplasm; (F) strong staining in cytoplasm; (G), (H), (I), (J) showing the higher magnification (200×) from the area of the box in (C), (D), (E) and (F), respectively.

We first evaluated the endogenous expression of SPARC in several human NPC cell lines and NPEC2 Bmi-1 cell line. To determine SPARC expression, quantitative real-time PCR was performed to evaluate SPARC mRNA expression levels in NPC cell lines including CNE1, CNE2, HONE1, SUNE1 and C666, and an immortalized primary nasopharyngeal epithelial cell line, NPEC2 Bmi-1. Compared to NPEC2 Bmi-1 cells, high expression levels of SPARC mRNA were observed in the NPC cell lines CNE1, CNE2, HONE1, SUNE1 and C666 (Figure 1A). Western blot analysis also revealed over-expression of SPARC protein in CNE1, CNE2, HONE1, SUNE1 and C666, compared to NPEC2 Bmi-1 (Figure 1B). Moreover, the expression of SPARC in the cell lines 5-8 F (a NPC cell line with high tumorigenic and metastatic ability) and 6-10B (a NPC cell line with high tumorigenic and low metastatic ability) were also analysed. Figure 1C-D showed that the expression levels of SPARC mRNA and protein in the 6-10B were lower than in the 5-8 F. The significance was assessed by *t*-test (*P *= 0.002, *P *= 0.001). The expression of SPARC protein was determined by IHC in NPC tissues. No staining was found in the stroma and the normal nasopharyngeal epithelial of NPC tissue (Figure 2A-B). Representative staining of SPARC was shown in Figure 2C-2J. SPARC immunostaining revealed cytoplasmic localization in NPC cells.

### SPARC expression and overall survival

**Figure 3:**
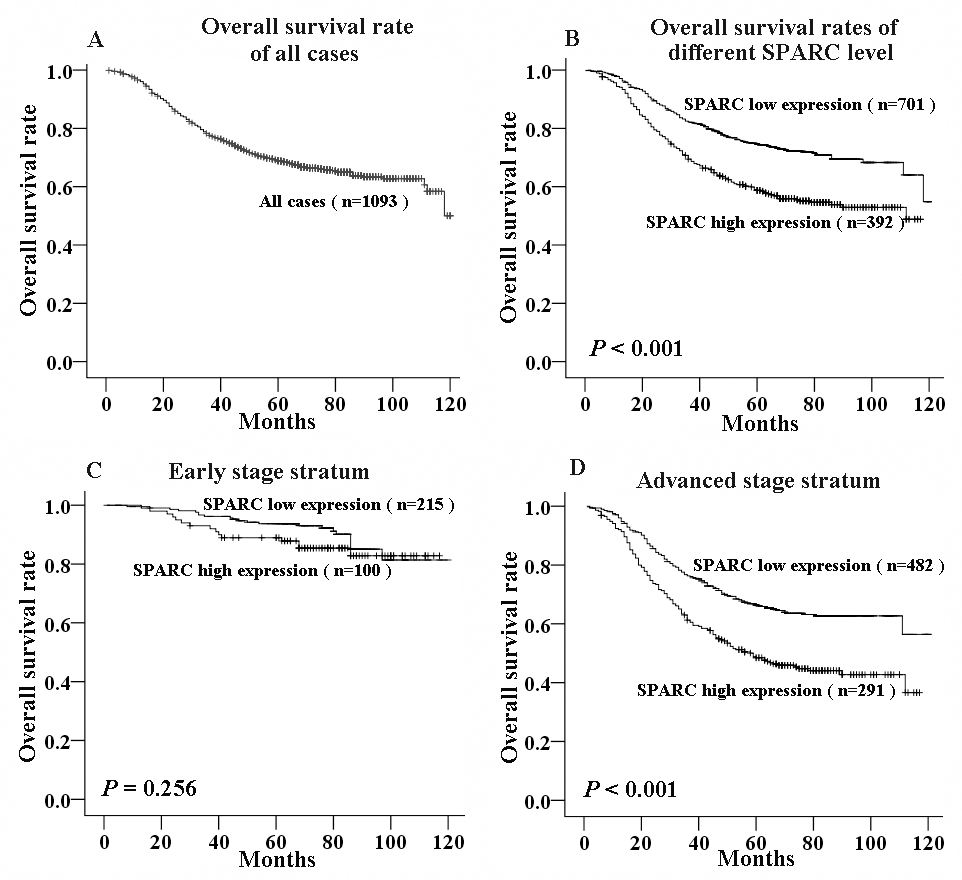
**Kaplan-Meier survival curve and log-rank test for NPC patients classified as showing either high or low SPARC expression**. (A) Kaplan-Meier curve for 5-year overall survival rate (69.1%) of 1093 patients with NPC; (B) Overall survival curve of NPC patients with different SPARC expression and the 5-year OS rate was significantly different between NPC patients with low expression (74.9%) and high expression (58.9%) (*P *< 0.001; log-rank test); (C) Cases stratified by clinical stage. Within the early stage (Stage I + II) stratum, SPARC expression did not show a statistical (*P *= 0.256; log-rank test) relationship with patients survival; (D) Within the advanced stage (Stage III + IV) stratum, SPARC expression exhibited a significant (*P *< 0.001; log-rank test) relationship with patients survival.

In the all NPC patients (1093 cases), the 5-year overall survival (OS) rate was 69.1% (Figure 3A). We defined 701 patients (64.1%) as low expression and 392 patients (35.9%) as high expression according to levels of SPARC expression. Among all 1093 patients, the 5-year OS rates differed substantially and statistically significantly between low expression and high expression patients (74.9% *vs*. 58.9%, Figure 3B; *P *< 0.001). After stratification by clinical stage, SPARC expression remained a significant predictor of NPC prognosis in the advanced stage (stages III-IV) but not a significant predictor in the early stage (stages I-II). In the early stage, the 5-year OS rate was 93.7% among low-expression patients, and 88.8% among high-expression patients (Figure 3C; *P *= 0.256). In addition, in the advanced stage, the 5-year OS rate was 66.8% among low-expression patients, and 48.8% among high-expression patients (Figure 3D; *P *< 0.001). The expression of SPARC also remained a clinical and statistical predictor of prognosis after stratification by WHO classification (*P *< 0.001), relapse (*P *< 0.001) and metastasis (*P *< 0.001) (data not shown).

### Association of SPARC with NPC patient's clinicopathological parameters and Cox Proportional Hazards Survival Analysis

The high or low expression rates of SPARC in NPC with respect to several standard clinicopathological features are presented in Table 2. There was a significant association between SPARC expression and distant metastasis of NPC (*P *= 0.001, Table 2), and WHO classification (*P *= 0.023, Table 2). There was no significant correlation between SPARC expression and other clinicopathological parameters, such as age, sex, clinical stage and relapse (*P *> 0.05, Table 2). Univariate Cox proportional hazard regression analysis revealed that high SPARC expression was the most significant predictive factor for poor prognosis of patients with NPC (*P *< 0.001, Hazard ration [HR] = 1.785). Other clinicopathological parameters, including age (*P *< 0.001, HR = 1.554), sex (*P *= 0.039, HR = 0.78), and clinical stage (*P *< 0.001, HR = 4.692) were also found to be predictive factors for poor prognosis of NPC patients (Table 3). The parameters that were significant in univariate analysis were further examined in multivariate analysis. After multivariate adjustment, high SPARC expression remained a powerful unfavorable predictor (*P *< 0.001, HR = 1.741) independent of other clinicopathological factors, including age (*P *< 0.001, HR = 1.551) and clinical stage (*P *< 0.001, HR = 4.766) (Table 3).

**Table 2:** Associations between SPARC expression and clinicopathologic characteristics among all NPC cases

SPARC expression				
			
Characteristics	Low (n = 701) (%)	High (n = 392) (%)	r	*P*-value
Age* (years)				
≤ 47	366 (52.5)	202 (52.1)		
> 47	331 (47.5)	186 (47.9)	0.004	0.887
**Sex**				
Female	209 (29.8)	109 (27.8)		
Male	492 (70.2)	283 (72.2)	0.021	0.483
**Clinical stage**				
I + II	215 (30.8)	100 (25.6)		
III + IV	482 (69.2)	291 (74.4)	0.056	0.066
**Relapse**				
No	649 (92.7)	352 (89.8)		
Yes	51 (7.3)	40 (10.2)	0.051	0.094
**Metastasis**				
No	647 (92.4)	337 (86.0)		
Yes	53 (7.6)	55 (14.0)	0.104	0.001
**WHO histological classification**				
NKUC	566 (80.9)	287 (73.8)		
NKDC	118 (16.9)	88 (22.6)		
KSCC	16 (2.2)	14 (3.6)	0.082	0.023

*Median age; *SPARC *secreted protein acidic and rich in cysteine; *NPC *nasopharyngeal carcinoma; *WHO *World Health Organization; *NKUC *nonkeratinized undifferentiated carcinoma; *NKDC *nonkeratinized differentiated carcinoma; *KSCC *keratinized squamous cell carcinoma

**Table 3:** Cox Regression analysis of the SPARC expression, clinicopathological variables for overall survival in NPC patients

Variable	Univariate Crude HR (95% CI)	*P *value	*Multivariate adjusted HR (95% CI)	*P *value
**SPARC expression**				
High *versus *Low	1.785 (1.455-2.190)	< 0.001	1.741 (1.414-2.144)	< 0.001
**Agec (years)**				
> 47 *versus *≤ 47	1.554 (1.265-1.910)	< 0.001	1.551 (1.259-1.910)	< 0.001
**Sex**				
Female *versus *Male	0.780 (0.616-0.987)	0.039	0.854 (0.673-1.083)	0.193
**Clinical Stage**				
III + IV *versus *I + II	4.692 (3.325-6.620)	< 0.001	4.766 (3.361-6.758)	< 0.001
**WHO histological classification**				
NKDC *versus *NKUC	0.935 (0.716-1.222)	0.625	0.919 (0.701-1.205)	0.54
KSCC *versus *NKUC	1.988 (1.183-3.342)	0.01	1.804 (1.069-3.047)	0.027

*Adjusted for SPARC expression, sex, WHO histological classification, age, and clinical stage. *SPARC *secreted protein acidic and rich in cysteine; *WHO *World health organization; *NPC *nasopharyngeal carcinoma; NKUC, non-keratinizing undifferentiated carcinoma; *NKDC *non-keratinizing differentiated carcinoma; *KSCC *keratinizing squamous cell carcinoma; *HR *hazard ratio; *CI *confidence interval

## Discussion

NPC is a malignant neoplasm arising from the mucosal epithelium of the nasopharynx, most often within the lateral nasopharyngeal recess, and is thought to be closely associated with Epstein-Barr virus infection, dietary, and genetic factors. The majority of NPC-related deaths are attributed to tumor metastasis rather than to the primary tumor. However, the molecular mechanisms underlying NPC invasion and metastasis are not completely understood. Thus, novel molecular markers that can identify tumor metastasis and aid in prognosis assessment are urgently needed.

Immunohistochemistry is an indispensable research tool frequently used to study tumor progression and prognosis. Here, we evaluated SPARC protein expression detected by immunohistochemical techniques in a large and well-documented cohort of primary NPC samples, and correlated the results with clinicopathological characteristics and patient survival. In many NPC specimens, over expression of SPARC was frequently detected. We also demonstrated that SPARC was highly expressed at both the mRNA and protein levels in NPC cell lines as compared with NPEC2 Bmi-1. The expression of SPARC in all normal nasopharyngeal epithelium detected by IHC was absent, suggesting that SPARC is a common feature in NPC that might play an important role in its prognosis and metastasis.

Over-expression of SPARC was frequently observed in the tumor specimens analyzed, and showed statistically significant association with high tumor metastasis and poor prognosis. In the patients here, higher SPARC expression was significantly associated with tumor progression (metastasis and poor prognosis) and the advanced stages of NPC. In addition, patients with lower SPARC expression had an improved prognosis. These observations between SPARC expression and tumor progression are consistent with other malignancies, such as gastric cancer [13] and renal carcinoma [35]. The expression of SPARC has been positively correlated with the histological grade of tumor cells in bladder cancer [21], thyroid cancer [36], glioma [37] and HCC [38]. In the present study, SPARC expression still remained a significant predictor in the advanced stage. Radiotherapy has become the standard treatment for NPC patients with earlier stage. Although chemo-radiotherapy is a popular therapy for advanced NPC, improving the survival of these patients still remains a significant challenge [39]. SPARC may be a marker for advanced NPC as a potential therapy agent. Advanced NPC patients with low SPARC expression may accept the mild treatment without the radical therapy. By contrast, advanced NPC patients with higher SPARC expression may benefit from higher-dose radiation, adjuvant therapy, or molecular target therapy. Multivariate Cox proportional hazards survival analysis suggested that SPARC over-expression had a significantly worse prognostic impact (*P *< 0.001) on survival of NPC patients. Consistent with the findings reported by the previous studies, we confirmed that the independently significant negative predictive factors for survival included advanced increased age (*P *< 0.001) and advanced clinical stage (*P *< 0.001) [40-42]. These results indicate that as an independent risk factor, SPARC may serve as a prognostic marker for survival of NPC patients.

SPARC functions as a regulator of cell-matrix interaction, and is generally recognized to mediate de-adhesion thereby promote cell migration [43]. It has a profound influence on cancer progression [44]. However, a previous study [45] revealed that SPARC expression was higher in NPEC than in NPC cell lines. With the results of the current study, we speculated that endogenous SPARC expression was higher in NPC cell lines than in the NPEC2 Bmi-1. This seems to contradict our current study. One possible reason is that the current results here were showed in a large retrospective cohort. Another reason may be the difference in the distribution of NPC patients and NPC cell types. Especially, high levels of SPARC often correlated with the lymph node metastasis, enhanced invasion, metastasis, and poor prognosis [13,17,46,47], for example, metastasis to the colon, lung, esophagus and pancreas [48]. Previous studies [49-51] using prostate cancer tissue samples reported that SPARC expression was higher in metastatic sites than in the primary site. These phenomena suggest that SPARC plays different roles in cancer progression in different tumor cell types and acts via different signal transduction pathways [52].

Our study may have suffered from the limitation: as discussed above the difference of SPARC expression between the NPEC2 Bmi-1 cell line and NPEC was not objectively verified. However, we chosen the NPEC2 Bmi-1 cell line as a control because it is the immortalized cell line closest to normal nasopharyngeal epithelium. Furthermore, the immortal NPEC cell line (NPEC2 Bmi-1) is a pre-malignant nasopharyngeal epithelial cell model and maintains a normal P53 checkpoint [53]. Compared with nasopharyngeal carcinoma cell lines, NPEC2 Bmi-1 cell line as a control may be feasible.

While a High SPARC level indicates poorer prognosis in some tumors, SPARC expression in neuroblastoma inhibits angiogenesis and impairs tumor growth [54]. For example, the increased SPARC expression in prostate cancer, bladder cancer [21], melanoma [24] and non-small cell lung cancer indicated a higher malignancy and invasion of tumors with poor prognosis. In contrast, in ovarian cancer, elevated SPARC expression inhibited the invasion and metastasis of tumor cells [30]. Thus, the varying influence of SPARC in different tumors reflects that the function of SPARC may be tissue-specific.

## Conclusions

In summary, SPARC plays a crucial role in the process of tumor invasion and metastasis in certain malignancies. Regardless of the underlying biological mechanism, SPARC expression status was proved to be of powerful prognostic predictive value in distinguishing patients with a more biologically aggressive and invasive nasopharyngeal carcinoma. The data provided by our study indicates that SPARC can serve as a useful biomarker to better determine NPC prognosis and appropriate therapeutic model. Further clinical and experimental studies are needed to define the genetic and/or epigenetic mechanisms leading to SPARC over-expression, and to better understand the role of SPARC in normal nasopharyngeal epithelium and NPC.

## Abbreviations

NPECs: Nasopharyngeal epithelial cells; IHC: Immunohistochemistry; SPARC: Secreted protein acidic and rich in cysteine; NPC: Nasopharyngeal carcinoma; WHO: World Health Organization; NKUC: Nonkeratinized undifferentiated carcinoma; NKDC: Nonkeratinized differentiated carcinoma; KSCC: Keratinized squamous cell carcinoma; TMA: Tissue microarray.

## Competing interests

The authors declare that they have no competing interests.

## Authors' contributions

JYS and YXZ are responsible for the study design. HYW and YYL carried out the experiments. HYW drafted the manuscript and participated in the data interpretation. QS, JHH, FW and MBC participated in the data collection and analysis. All authors read and approved the final manuscript.
